# 중환자실 간호사를 위한 QR-Code를 활용한 동영상 기반 경장영양 교육 프로그램의 효과

**DOI:** 10.7586/jkbns.24.001

**Published:** 2024-02-23

**Authors:** Won Kee Seo, Hyunjung Kim

**Affiliations:** 1 Department of Nursing, Seoul National University Bundang Hospital, Seongnam, Korea; 1 분당서울대학교병원 간호본부; 2 Graduate School of Nursing Science, Hallym University, Chuncheon, Korea; 2 한림대학교 간호대학원; 3 School of Nursing, Research Institute of Nursing Science, Hallym University, Chuncheon, Korea; 3 한림대학교 간호대학, 간호학연구소

**Keywords:** Education, Enteral nutrition, Intensive care units, Nurses, 교육, 경장영양, 중환자실, 간호사

## Abstract

**Purpose:**

This study aimed to investigate the effect of a video-based enteral nutrition education program using QR codes on the perception, knowledge, and performance of enteral nutrition for intensive care unit (ICU) nurses.

**Methods:**

This was a quasi-experimental study with a nonequivalent control group pre- and post-test design. The participants were nurses working at six ICUs within a single university hospital, with 55 nurses in the experimental group and 55 nurses in the control group. The video-based enteral nutrition education program using QR codes was repeated three times to the experimental group.

**Results:**

There was a statistically significant pre-post difference in perceptions between the two groups (Z = -4.04, *p* < .001) with 2.00 points (± 5.57) for the control group and 7.89 points (± 7.95) for the experimental group, in knowledge (Z = -7.48, *p* < .001) with 0.02 points (± 1.91) for the control and 4.18 points (± 2.33) for the experimental, and in performance (Z = -2.20, *p* = .028) with 0.06 points (± 3.96) for the control and 2.00 points (± 5.14) for the experimental.

**Conclusion:**

The video-based enteral nutrition education program using QR codes was effective in improving the perceptions, knowledge, and performance of enteral nutrition among ICU nurses. This enteral nutrition education program using QR codes in clinical education can contribute to evidence-based nursing practice by improving perceptions and knowledge of enteral nutrition.

## 서론

### 1. 연구의 필요성

중환자실 환자 중 대다수는 손상, 쇼크, 패혈증 및 다기관부전 등의 상태로 인하여 대사량과 단백질 이화 작용이 증가한다[1]. 국외의 18세 이상 중환자를 대상으로 한 체계적 문헌 고찰을 통한 분석 결과에 따르면 중환자의 37.8%-78.1%가 영양불량 상태이었다[2]. 국내에서 중환자를 대상으로 총림프구수, 혈중 알부민 농도를 측정하여 영양 상태를 평가한 결과에 따르면 중환자의 68.3%가 영양불량 상태이었다[3]. 그러나 중환자들에게 영양이 적절히 충족되지 못하면 영양불량뿐만 아니라 면역기능 저하로 감염과 같은 다양한 합병증을 일으킨다[4].

중환자에게는 경장영양 또는 정맥영양 경로를 통해 영양지원을 할 수 있는데, 금기 사항이 아닌 경우 중환자들에게 경장영양을 통한 영양지원이 우선시 되고 있다[5]. 간호사는 환자에게 제공되는 영양 지원 방법의 적정성을 평가하고, 영양 공급을 시작하고 관리하며, 잠재적 또는 실재적으로 발생할 수 있는 합병증에 대하여 평가를 하는 등의 중요한 역할을 담당한다[6,7]. 그러나 실제 임상 현장에서 간호사들은 검사 및 시술 진행, 투약 및 처치, 응급 상황의 대처 등으로 인해 경장영양을 간호중재의 우선순위에서 제외하고 있으며, 궁극적으로 중환자에게 경장영양이 적절하게 제공되지 못하게 하고 있다[8]. 그러므로 중환자에게 효과적으로 영양을 제공하기 위해서는 간호사에게 근거 기반의 경장영양 지원 프로토콜의 제공이 필요하다[9]. 일시적인 교육 프로그램보다는 지속적인 교육을 제공하여 실무에서 적용할 수 있도록 함으로써 간호사들의 중환자 영양 관리 능력의 향상을 기대할 수 있을 것이다[10].

영양교육 프로그램을 간호사에게 적용하여 중재하는 국내외 연구를 살펴보면, 간호사에게 대면 교육 및 동료 관찰 등을 포함한 영양 교육 프로그램을 통해 영양에 대한 인식이 향상됨을 보였고[11], 중환자실 간호사를 대상으로 대면 강의 방식을 통한 경장영양 교육 프로그램을 적용하여 경장영양에 대한 인식, 지식이 향상되는 결과를 나타내었으며[12], 경장영양에 대한 수행도가 크게 상승하였음을 보였다[13]. 간호사에게 경장영양에 대한 교육을 제공하는 것은 간호사들의 경장영양에 대한 지식과 수행도를 향상시켜 환자에게 안전하게 경장영양을 공급하게 하므로 간호사들에게 경장영양에 대한 지속적인 교육은 반드시 필요하다[14].

전통적인 오프라인 교육을 제공하기 위해서는 간호사들의 교대 근무로 인해 참여가 제한되는 것과는 달리 온라인 교육은 학습 시간 절약과 비용효과성을 기대할 수 있다[15]. 온라인 교육에서 동영상을 활용한 학습 자료는 영상과 더불어 음향에 의한 자극까지 더해지기 때문에 인쇄 매체나 다른 매체를 활용할 때보다 학습자의 주의 집중력, 설명 능력, 기억 유지 능력 등을 함양시킬 수 있는 기능을 지니고 있다[16]. 그러나 기존의 동영상을 활용한 온라인 교육은 고정된 컴퓨터의 웹사이트를 통해서 접근이 가능하도록 하여 공간적인 제한이 있다[17]. 스마트폰 보급화가 진행됨에 따라 컴퓨터에서 온라인 교육에 접근하는 방식을 넘어서, 시간과 공간의 제약 없이 다양하고 풍부한 지식을 용이하게 접근하여 학습 경험을 확장시키고 있다[18]. 이에 스마트폰 어플리케이션을 활용한 교육이 시도되어 왔으나 이 방법은 어플리케이션 프로그램의 설치와 구동 시간에서 제한점을 가지고 있다[19]. 최근에는 스마트폰으로 2차원 바코드인 ‘QR-Code’를 인식하여 교육 자료에 바로 접근하게 함으로써 교육의 효과를 확인하는 연구들이 보고되고 있다. QR-Code를 이용한 학습은 편의성이 높은 장점이 있으며, 학습자의 정보 검색에 동기를 부여할 뿐만 아니라 호기심과 탐구심을 자극할 수 있다[20]. QR-Code를 교육 목적으로 활용한 선행 연구를 구체적으로 살펴보면 의학 분야에서는 QR-Code를 활용하여 정형외과 수술 후 보조기 착용법에 대한 교육을 받은 환자들이 구두로 설명을 받은 환자들에 비해 보조기 착용 능력과 이해도 측면에서 더 높은 결과를 보였다[21]. 간호학에서는 QR-Code를 활용한 기본간호학 실습이 간호 학생의 근육주사 수행 자기효능감을 유의하게 상승시켰고[17], QR-Code를 이용한 자가간호 교육이 기흉 환자의 자기효능감, 자가간호 수행, 교육 만족도를 높였다[18]. 그리고 관상동맥 조영술 환자에게 QR-Code를 활용한 동영상 교육을 적용하였을 때 시술 전 준비사항과 시술 후 주의사항에 대한 순응도가 높게 나타났다[22]. 이와 같이 여러 분야에서 QR-Code를 활용한 교육의 효과를 보고하고 있다.

중환자의 회복에 큰 영향을 끼치는 영양 치료인 경장영양을 적절히 공급하기 위해서는 경장영양을 실제로 수행하는 간호사에게 경장영양 교육을 적용하는 것이 효과적이다는 점을 알 수 있었다. QR-Code를 활용한 교육이 효과적임을 보여주는 연구들이 보고되고 있으나, 임상 현장에 근무하는 간호사를 대상으로 QR-Code를 활용한 교육 프로그램을 적용한 연구는 거의 없다. 교대 근무와 높은 업무 강도로 인해 오프라인 교육이 어려운 간호사에게 스마트폰으로 QR-Code를 인식하면 교육 프로그램으로 바로 연결이 되는 방법은 교육의 접근성과 편의성을 증진시킬 것으로 기대된다. 이에 QR-Code와 동영상을 접목한 경장영양 교육 프로그램을 제공하여 스마트폰으로 시공간의 제약 없이 반복 학습할 수 있게 함으로써 교육 프로그램의 효과를 파악하고자 한다. 이를 통해 다양한 임상 환경에 있는 간호사들에게 경장영양 및 영양 교육 프로그램을 개발하여 제공하기 위한 기초 자료를 제공하고자 한다.

### 2. 연구의 목적

본 연구의 목적은 중환자실 간호사를 대상으로 QR-Code를 활용한 동영상 기반 경장영양 교육 프로그램을 적용하여 경장영양에 대한 인식, 지식, 수행도에 미치는 효과를 규명하기 위함이다. 연구의 가설은 다음과 같다.

· 제1가설: QR-Code를 활용한 동영상 기반 경장영양 교육 프로그램을 적용한 실험군은 교육을 받지 않은 대조군보다 경장영양에 대한 인식이 높을 것이다.

· 제2가설: QR-Code를 활용한 동영상 기반 경장영양 교육 프로그램을 적용한 실험군은 교육을 받지 않은 대조군보다 경장영양에 대한 지식이 높을 것이다.

· 제3가설: QR-Code를 활용한 동영상 기반 경장영양 교육 프로그램을 적용한 실험군은 교육을 받지 않은 대조군보다 경장영양에 대한 수행도가 높을 것이다.

## 연구 방법

### 1. 연구 설계

본 연구는 중환자실 간호사를 대상으로 QR-Code를 활용한 동영상 기반 경장영양 교육 프로그램을 총 3회 반복 적용하여 중환자실 간호사의 경장영양에 대한 인식, 지식, 수행도의 변화를 파악하고, 교육 프로그램의 효과를 분석하는 비동등성 대조군 사전·사후 설계 유사 실험 연구이다.

### 2. 연구 대상

본 연구는 2023년 경기도에 소재한 분당서울대병원에 근무 중인 내과계중환자실, 외과계중환자실, 뇌신경계중환자실, 응급중환자실1, 응급중환자실2, coronavirus disease-2019 (COVID-19) 중환자실을 포함한 총 6개 중환자실 간호사를 대상으로 편의 표집하였다. 대상자 수의 선정은 G-power 3.1 프로그램을 이용하여 independent t-test, 양측 검정, 선행연구[12]에서의 효과크기 .61, 유의수준 .05, 검정력 .80으로 필요한 적정 수의 표본크기를 산출한 결과 각 집단 45명, 총 90명이 산출되었다. 본 연구에서는 탈락률 30%를 고려하여 집단별 63명을 계획하였다.

실험의 확산 효과가 우려되어 실험군과 대조군은 중환자실 배정을 다르게 하였다. 실험군은 내과계중환자실, 외과계중환자실, 응급중환자실1, COVID-19 중환자실로 배정하였고, 대조군은 뇌신경계중환자실, 응급중환자실2로 배정하였다. 각 중환자실마다 주요 진료과 및 환자 질병의 특성상 평균 환자 재실 기간이 다름을 고려하여, 상대적으로 장기 재실 환자가 많은 내과계중환자실과 뇌신경계중환자실을 각각 실험군과 대조군으로 배정하였다. 상대적으로 단기 재실 환자가 많은 응급중환자실과 응급중환자실2를 각각 실험군과 대조군에 배정하였다. 또한 각 중환자실별 간호사 인력 규모를 고려하여 실험군과 대조군의 모집단 규모를 최대한 유사하게 설계하기 위하여 외과계중환자실과 COVID-19 중환자실을 실험군과 대조군으로 나누지 않고 모두 실험군에 배정하였다.

**Figure 1.**
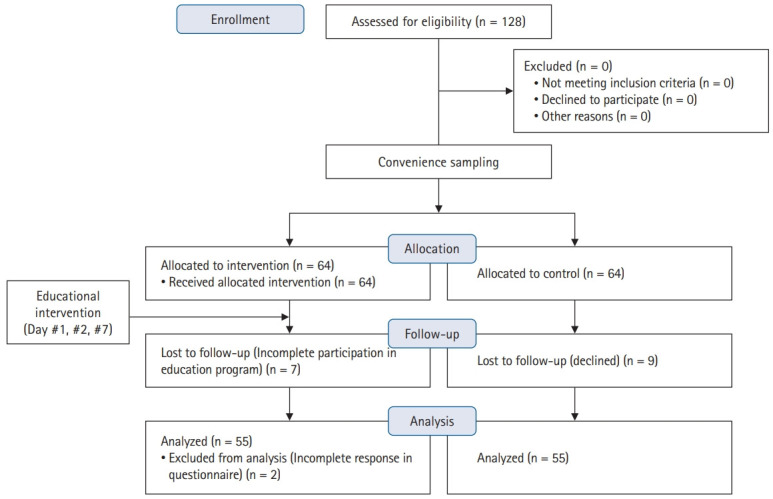
Research flow diagram.

사전 설문 조사에 실험군 64명, 대조군 64명이 참여하였고, 모두 선정기준에 부합하여 실험군 교육 중재에 64명이 모두 포함되었다. 실험군 중 7명과 대조군 중 9명이 중도 탈락하여 사후 설문 조사에 실험군 57명, 대조군 55명이 참여하였고, 실험군 사후 설문 조사 중 2명의 설문지 응답이 미비하여 제외하였다. 이에 최종적으로 실험군 55명, 대조군 55명이 본 연구의 분석에 포함되었다(Figure 1).

### 3. 연구 도구

#### 1) 중환자실 간호사의 경장영양에 대한 인식

Persenius 등[23]이 개발한 경장영양에 대한 간호사들의 인식 도구를 한국어로 번역한 도구[24]를 사용하였다. 이 도구는 경장영양에 대한 책임 영역 5문항, 지식 영역 5문항, 기록 영역 5문항, 총 3개 영역 15문항으로 구성되어 있다. 책임 영역은 본인이 어느 정도의 책임을 가지고 있다고 생각하는가를, 지식 영역은 본인이 어느 정도의 지식을 가지고 있다고 생각하는가를, 기록 영역은 기록이 어느 정도 필요하다고 생각하는가를 자가 평가하도록 구성되어 있다. 각 문항은 5점 Likert scale이며, 점수 범위는 최저 15점에서 최고 75점으로 점수가 높을수록 경장영양에 대한 인식이 높은 것을 의미한다. Kim과 Soun [24]의 연구에서 Cronbach’s α는 책임 .87, 지식 .88, 기록 .91이었다. 본 연구에서의 Cronbach’s α는 .87, .92, .87이었고, 총 Cronbach’s α는 .93이었다.

#### 2) 중환자실 간호사의 경장영양에 대한 지식

American Society for Parenteral and Enteral Nutrition [25]과 European Society for Parenteral and Enteral Nutrition [26]에서 권장하는 경장영양 공급 가이드라인에 기초하여 Kim과 Soun [24]이 개발한 20문항의 도구를 일부 수정하여 사용하였다. 본 연구에서는 문항 신뢰도가 낮은 영양공급의 평가 1문항을 제외한, 총 19문항으로 측정하였다. 정답은 1점, 오답은 0점으로 점수화하였고, 최저 0점에서 최고 19점으로 점수가 높을수록 지식이 높은 것을 의미한다. Kim과 Soun [24]의 연구에서 Kuder-Richardson Formula 20 (KR 20)은 .64이었으며, 본 연구에서의 KR 20은 .61이었다.

#### 3) 중환자실 간호사의 경장영양에 대한 수행도

Persenius 등[23]이 개발한 경장영양에 대한 간호사들의 수행도 도구를 한국어로 번역한 도구[24]에서 최신 지견 및 최근의 임상 상황과 다소 차이가 있는 간호 행위 1문항을 제외한, 총 14문항으로 측정하였다. 각 문항은 5점 Likert scale이며, 점수 범위는 최저 14점에서 최고 70점으로 점수가 높을수록 경장영양에 대한 수행도가 높은 것을 의미한다. Kim과 Soun [24]의 연구에서 Cronbach’s α는 .68, 본 연구에서의 Cronbach’s α는 .74이었다.

### 4. QR-Code를 활용한 동영상 기반 경장영양 교육 프로그램 구성

본 교육 프로그램은 Kim [27]이 개발한 ‘동영상 기반 중환자 영양 지원 교육 프로그램’을 바탕으로 연구대상자의 소속 병원 실정에 해당하지 않는 세부 내용을 삭제하고, 소속 병원에 해당하는 내용으로 일부 변경하여 총 9개의 영역, 14분 16초 분량으로 구성하였다.

내용 타당성 검증을 위해 간호학 교수 3인, 중환자실 수간호사 6인, 외과 전문의 1인, 영양사 1인으로 구성된 전문가 집단 총 11인에게 프로그램의 목적 및 내용에 대한 항목별 내용타당도(item content validity index, I-CVI) 검증을 받았다. 모든 영역의 CVI가 .80 이상으로 제외되어야 하는 항목은 없었으며, scale-CVI/Ave는 .99이었다. 동영상 내용의 난이도 및 음질·화질 상태 등을 전반적으로 점검하기 위해 간호학 석사 학위를 소지하고 임상 경력 10년 이상의 중환자실 간호사 5명을 대상으로 적합성 평가를 실시하였다. 각 문항 4점 척도에서 10개의 모든 항목이 평균 3.6점 이상으로 높은 적합성을 보였다.

### 5. 자료 수집 절차

#### 1) 사전 조사

2023년 3월 20일부터 3월 23일까지 대조군과 실험군의 사전 조사가 진행되었다. 2023년 3월 20일에 연구자가 직접 대조군과 실험군의 근무 부서로 방문하여 부서 게시판에 연구대상자 모집 공고문을 게시하고, 부서 스테이션에 연구대상자 설명문 및 동의서와 사전 조사 설문지를 비치하고 회수함을 설치하였다. 연구에 자발적으로 참여하고자 하는 간호사는 연구대상자 동의서와 사전 조사 설문지를 작성한 후 밀봉하여 회수함에 보관하도록 한 후 연구자가 직접 수거하였다.

#### 2) 교육 중재

실험군에게는 개인의 스마트폰으로 QR-Code를 활용한 동영상 기반 경장영양 교육 프로그램을 시청하도록 동영상 접근 방법을 설명하는 연구대상자 설명문을 개별적으로 제공하였다. 설명문에 나와 있는 QR-Code를 인식하여 동영상 기반 경장영양 교육 프로그램에 접근하여 시청하도록 하였다. 안내지에는 3월 24일, 3월 25일, 3월 30일 총 3회 개별적으로 동영상을 꼭 시청하여야 한다는 내용을 포함하였다. 연구자는 실험군에게 개별적으로 실험 처치 당일인 3월 24일, 3월 25일, 3월 30일에 문자메시지를 전송하여 동영상 시청을 꼭 하도록 안내하였다. Ebbinghaus의 망각 주기 이론에 근거한 주기학습법[28]을 적용하여 망각 시점인 1일·2일·7일·30일의 총 4회로 구성된 반복 혈액투석 식이 교육 프로그램을 적용한 선행연구[29]에 근거하여, 본 연구에서는 1일·2일·7일의 총 3회의 교육 프로그램을 제공하였다.

#### 3) 사후 조사

2023년 3월 30일부터 4월 2일까지 실험군의 총 3회의 교육 프로그램 적용이 완료된 직후 대조군과 실험군의 사후 조사가 진행되었다. 2023년 3월 30일에 연구자가 직접 대조군과 실험군의 근무 부서로 방문하여 부서 스테이션에 사후 조사 설문지를 비치하고 회수함을 설치하였다. 설문지를 작성한 후 밀봉하여 회수함에 보관하도록 한 후 연구자가 직접 수거하였다.

### 6. 자료 분석 방법

수집된 자료는 SPSS/WIN 25.0 통계 프로그램(IBM Corp., Armonk, NY, USA)을 이용하여 분석하였다. 대상자의 일반적 특성은 기술 통계 방법을 이용하여 실수와 백분율을 산출하였다. 대조군과 실험군의 일반적 특성에 대한 동질성 검증은 χ²-test, Fisher's exact test를 이용하여 분석하였으며, 종속변수에 대한 사전 정규성 검정을 모두 충족하지 않았기에 종속변수에 대한 사전 동질성 검증은 Mann-Whitney U test로 분석하였다. 교육 프로그램의 효과에 대한 가설 검정은 Wilcoxon’s signed-ranks test로 분석하였다. 본 연구의 통계적 유의수준은 *p* < .05에서 검정하였다.

### 7. 윤리적 고려

본 연구는 연구대상자의 보호를 위해 사전에 연구대상자가 속한 분당서울대병원 기관생명윤리위원회의 승인(IRB No. B-2301-804-304)을 받은 후 시행하였다. 자료수집에 앞서 연구자가 간호본부장의 결재 승인으로 기관의 허락을 받았다. 연구자는 연구대상자 설명문을 통해 연구대상자에게 본 연구에 참여하는 것은 자율 의지에 따르며, 연구 참여 도중 원하지 않을 경우 언제든지 참여에 중단할 수 있으며, 중단 후 어떠한 불이익도 받지 않는 점을 알렸다. 그리고 연구대상자의 개인정보에 관한 기록은 비밀로 보호될 것임과 모든 결과는 익명으로 코드화하여 처리하며 연구 이외의 어떤 목적으로도 이용되지 않음을 고지하였다. 이와 같은 설명문을 읽고 연구대상자 동의서에 자필 서명한 대상자들은 연구에 참여하였다. 또한 본 연구의 실험군에게만 QR-Code를 활용한 동영상 기반 경장영양 교육 프로그램을 제공하는 경우 윤리적 문제가 있을 수 있어 대조군에게도 실험군의 중재 적용이 모두 끝난 이후 실험군과 동일한 프로그램을 제공하였다. 또한 대조군과 실험군 모두에게 소정의 기프티콘을 제공하여 연구 참여에 대한 감사를 표시하였다.

## 연구 결과

### 1. 대상자의 일반적 특성 및 대조군과 실험군 동질성 검정

본 연구의 대상자의 연령은 25~29세(50.9%)가 과반수 이상이었고, 최종 학력은 학사(90.9%)가 대부분이었다. 중환자실 근무경력은 5년 미만이 62.8%이었으며, 그 중 2년 미만이 28.2%이었다. 대조군과 실험군의 연령, 성별, 최종학력, 직위, 총 임상 경력, 중환자실 근무경력, 경장영양 교육 경험은 모두 유의한 차이가 없었으므로 두 집단은 동질하였다(Table 1).

### 2. 대조군과 실험군의 경장영양에 대한 인식, 지식, 수행도 및 사전 동질성 검정

사전 동질성 검증에서 경장영양에 대한 인식은 75점 만점에 대조군 50.49점(± 9.25), 실험군 51.27점(± 6.89)으로 실험군이 높지만, 통계적으로 유의한 차이는 없었고(*p* = .409). 경장영양에 대한 지식은 19점 만점에 대조군 8.69점(± 1.85), 실험군 8.75점(± 1.88)으로 실험군이 높지만, 통계적으로 유의한 차이는 없었다(*p* = .745). 그러나 경장영양에 대한 수행도는 70점 만점에 대조군이 59.36점(± 6.46), 실험군이 62.22점(± 5.24)으로 실험군이 높고, 통계적으로 유의하여(*p* = .009), 두 집단은 인식과 지식에서는 사전 동질성이 확보되었으나 수행도에서는 사전 동질성이 확보되지 않았다(Table 2). 이에 대조군과 실험군의 프로그램 사전·사후 차이 평균을 비교하여 프로그램의 효과를 분석하였다.

### 3. 대조군과 실험군의 경장영양에 대한 인식, 지식, 수행도 사전·사후 차이 평균 비교 및 가설검증

1) 제1가설: ‘QR-Code를 활용한 동영상 기반 경장영양 교육 프로그램을 적용한 실험군은 적용하지 않은 대조군보다 경장영양에 대한 인식이 높을 것이다’는 대조군과 실험군의 인식 사전·사후 차이 평균은 대조군 2.00점(± 5.57), 실험군 7.89점(± 7.95)으로 두 군 간에 통계적으로 유의한 차이가 있었다(Z = -4.04, *p* < .001). 이에 제1가설은 지지되었다. 하부 카테고리인 책임(Z = -3.04, *p* = .002), 지식(Z = -4.57, *p* < .001), 기록(Z = -2.25, *p* = .024)의 분석에서도 사전·사후 차이 평균이 두 군 간에 모두 유의한 차이가 있었다.

2) 제2가설: ‘QR-Code를 활용한 동영상 기반 경장영양 교육 프로그램을 적용한 실험군은 적용하지 않은 대조군보다 경장영양에 대한 지식이 높을 것이다’는 대조군과 실험군의 지식 사전·사후 차이 평균은 대조군 0.02점(± 1.91), 실험군 4.18점(± 2.33)으로 두 군 간에 통계적으로 유의한 차이가 있었다(Z = -7.48, *p* < .001). 이에 제2가설은 지지되었다.

3) 제3가설: ‘QR-Code를 활용한 동영상 기반 경장영양 교육 프로그램을 적용한 실험군은 적용하지 않은 대조군보다 경장영양에 대한 수행도가 높을 것이다’는 대조군과 실험군의 수행도 사전·사후 차이 평균은 대조군 0.06점(± 3.96), 실험군 2.00점(± 5.14)로 두 군 간에 통계적으로 유의한 차이가 있었다(Z = -2.20, *p* = .028). 이에 제3가설은 지지되었다(Table 3).

## 논의

본 연구는 중환자실 간호사를 대상으로 QR-Code를 활용한 동영상 기반 경장영양 교육 프로그램을 적용하여 중환자실 간호사의 경장영양에 대한 인식, 지식, 수행도에 미치는 효과를 검증하고자 시도되었다.

실험군과 대조군의 경장영양에 대한 인식, 지식, 수행도의 사전 동질성 검정에서 인식과 지식은 사전 동질성이 확보되었으나, 수행도에서 사전 동질성이 확보되지 않아 각 군의 사전·사후 차이 평균을 비교하여 QR-Code를 활용한 동영상 기반 경장영양 교육 프로그램의 효과를 분석하였다.

본 연구에서 QR-Code를 활용한 동영상 기반 경장영양 교육 프로그램 중재를 받은 실험군의 경장영양에 대한 인식이 교육을 받지 않은 대조군보다 향상되어 교육 프로그램의 효과가 있었음을 확인하였다. 이와 같은 결과는 QR-Code를 활용한 선행연구가 전무하여 직접적인 비교는 어려우나, 중환자실 간호사를 대상으로 대면 경장영양 교육을 적용한 후에 인식 수준이 상승한 연구[12]의 결과를 뒷받침하고 있다. 그러나 본 연구대상자의 본인의 지식 정도에 대한 인식은 사전에도 가장 낮은 결과를 보였으나, 사후에도 경장영양의 책임과 기록의 필요성에 대한 인식보다 상대적으로 낮은 점수를 유지하였다. 이는 선행연구[12]와도 유사한 경향을 보이고 있다. 간호 대상자에게 적절한 경장영양을 제공하기 위해서는 간호사의 경장영양에 대한 높은 인식이 요구되므로[10], 간호사에게 지속적인 교육을 제공하여 인식을 제고하는 것이 필요하다.

제2가설인 ‘QR-Code를 활용한 동영상 기반 경장영양 교육 프로그램을 적용한 실험군은 교육을 받지 않은 대조군보다 경장영양에 대한 지식이 높을 것이다’는 지지되어 교육 프로그램의 지식에 대한 효과를 입증하였다. 이는 중환자실 간호사를 대상으로 대면 경장영양 교육을 적용한 후에 지식 수준이 상승한 선행 연구[12]의 결과와 유사하였다. 이러한 간호사들의 경장영양에 대한 낮은 지식 점수는 지속적으로 보고되어온 문제이다[12,24,25]. 본 연구와 동일한 도구를 사용한 선행연구[12]에서의 사전 지식점수가 20점 만점에 9.7점이었던 것과 본 연구에서의 사전 지식 점수가 유사한 결과가 이를 지지한다. 국내 중환자실 간호사들은 최신의 과학적 근거를 반영한 영양 정보가 제공됨에도 불구하고, 주로 동료들로부터 얻는 영양에 대한 정보에 의존하는 경향이 있다 [25,30]. 본 연구에서도 경장영양 교육을 한 번도 받아 본 경험이 없는 중환자실 간호사들이 약 1/3에 해당하였다. 경장영양 교육을 받은 경험이 있는 간호사들도 교육을 받게 된 경로 중 간호학부 수업이 절반을 차지하였고, 학부 졸업 이후 임상 현장에서 근무하면서 원내 교육이나 학회 등을 통해서 최신 지견을 교육 받은 비율은 낮게 나타났다. 이러한 경장영양 교육 참여의 부족, 특히 최신 근거에 기반하여 업데이트 된 교육에의 참여 부족이 간호사들의 경장영양에 대한 지식 부족에 기여하는 것으로 여겨진다. 간호사의 영양공급에 대한 지식 및 교육의 부족은 영양을 충분하게 제공하지 못하는 하나의 원인이 되고 있으며, 이는 중환자들의 영양실조로 이어지고 잠재적으로 환자 치료를 저해하게 된다[31]. 그러므로 임상에서 근무하는 중환자실 간호사들에게 최신 지견과 임상 상황을 반영한 경장영양 교육 제공이 주기적으로 필요하며[30], 이는 궁극적으로 환자들의 건강 상태 호전을 도모할 수 있을 것으로 기대된다.

제3가설인 QR-Code를 활용한 동영상 기반 경장영양 교육 프로그램 중재를 받은 실험군의 경장영양에 대한 수행도가 교육을 받지 않은 대조군보다 교육 후에 향상되어 교육 프로그램의 효과가 있었음을 확인하였다. 이와 같은 결과는 중환자실 간호사를 대상으로 대면 경장영양 교육을 적용한 후에 수행도 수준이 상승한 선행 연구[12,13]의 결과와 유사하였다. 본 연구에서의 실험군 내에서의 경장영양에 대한 수행도 점수 변화를 분석한 결과 사후가 사전보다 유의하게 점수가 상승한 문항보다 상승하지 않은 문항이 더 많았다. 이는 교육 중재를 받은 기간이 1주일이었고 중재가 끝난 직후 수행도를 평가하였기에 인식이나 지식과는 달리 행동의 변화를 보이기에는 부족한 시간이었다고 여겨진다. 또한 본 연구가 진행된 간호사 대 환자의 비율이나 간호사의 업무 부담감과 같은 근무 환경의 변화가 없는 상황에서 수행도의 급격한 변화를 유발하기에는 어려움이 있을 수 있다[32]. 그러므로 추후 연구에서는 수행도에 대한 교육 직후 효과 뿐만 아니라 장기적인 효과를 평가하는 것이 필요하다. 근거 기반 가이드라인에 따라 지속적인 경장 영양 교육을 계획하는 것이 중환자실에서 경장영양 수행도를 높이는 데에 필요하므로[33], 중환자실 간호사들에게 경장영양 교육은 꾸준하게 반복되어야 한다.

이와 같이 중환자실 간호사를 대상으로 스마트폰으로 인식하는 QR-Code와 동영상을 활용한 경장영양 교육 프로그램을 접목하여 시도한 본 연구를 통해 중환자실 간호사의 경장영양에 대한 인식과 지식 및 수행도를 향상시키는 효과가 입증되었다. 그러므로 시공간의 제약 없이 반복 학습 할 수 있는 QR-Code를 활용한 동영상 교육을 간호사 교육에 적극적으로 활용할 필요가 있다.

본 연구에는 몇 가지 제한점이 있다. 먼저, 본 연구는 연구 대상으로 6개 중환자실 간호사를 무작위 배정하지 않고 임의로 실험군과 대조군을 선정하여 자료수집을 시행하였으므로 연구의 결과를 일반화하는 것에 제한점이 있다. 또한, 본 연구에서 수행도에 대한 사후 조사를 교육 중재가 끝난 후 충분한 기간이 지난 후 측정하지 않아 시험 효과에 주의를 기해야 한다. 더욱이, 실험군이 실제로 QR-Code를 활용한 동영상 기반 경장영양 교육 프로그램을 총 3회 시청하였는지 정확히 확인할 수 없으므로 시청 횟수를 확인할 수 있는 방법이 필요하다.

그럼에도 불구하고, 본 연구는 교대 근무와 높은 업무 강도의 특성상 근무 외에 학습에 집중할 수 있는 여건이 비교적 어려운 경향이 있는 중환자실 간호사들에게 경장영양에 대한 학습을 시공간의 제약 없이 학습할 수 있는 QR-Code를 활용한 교육 프로그램을 적용하였다는 점에서 의의가 있다. 또한 본 연구에서 교육 자료는 동영상 기반으로 개발하여 제공하였으며, 이러한 동영상 교육은 학습자의 시각과 청각을 자극하여 실제 경험과 가까운 학습 경험을 제공하는 매체로서 학습자가 원하는 시간과 장소에서 교육이 가능하도록 하므로[34], 간호사를 위한 적합한 교육 방법으로 여겨진다.

## 결론

본 연구 결과, QR-Code를 활용한 동영상 기반 경장영양 교육 프로그램을 적용한 실험군의 경장영양에 대한 인식, 지식 및 수행도가 대조군에 비해 모두 높았다. 이는 QR-Code를 활용한 동영상 기반 경장영양 교육 프로그램이 경장영양에 대한 인식, 지식 및 수행도를 향상시키는 효과가 있었음을 보여준다. 본 연구는 임상에서 근거 기반 경장영양 및 영양 교육 프로그램을 개발하고 제공하기 위한 기초 자료로 활용될 것으로 사료된다. 궁극적으로는 중환자실 간호사들의 효과적인 경장영양 간호를 통해 중환자들의 영양 상태 향상에 기여할 것으로 기대한다. 나아가, 본 연구에서 사용한 QR-Code를 활용한 동영상 기반 경장영양 교육 프로그램을 각 병원의 실정에 맞게 수정하여 중환자실 간호사뿐만 아니라 병동 간호사 등을 대상으로 하여 교육 중재 및 효과를 파악하는 반복 연구가 필요하다. 또한 교육 프로그램의 효과가 얼마동안 지속되는지를 평가함으로써 효과적인 교육의 주기를 확인할 수 있는 장기적인 관찰 연구를 제언한다. 임상에서는 원내 교육이나 보수 교육을 통해 간호사들의 최신 근거에 기반한 영양 공급 교육을 주기적으로 제공하여 간호사들의 지식을 업데이트하고 인식과 수행도를 유지·향상시키는 것이 필요하다.

## Figures and Tables

**Table 1.** Characteristics of Study Participants and Homogeneity Test (N = 110)

Characteristics	Categories	Total (n = 110)		Control group (n = 55)		Experimental group (n = 55)		X^2^	*p*
		n	%	n	%	n	%		
Age (yr)^†^	≤ 24	9	8.2	5	9.1	4	7.3	3.15	.422
	25–29	56	50.9	30	54.5	26	47.3		
	30–39	42	38.2	20	36.4	22	40.0		
	≥ 40	3	2.7	0	0.0	3	5.5		
Sex	Female	89	80.9	45	81.8	44	80.0	0.06	.808
	Male	21	19.1	10	18.2	11	20.0		
Education^†^	Diploma	3	2.7	1	1.8	2	3.6	0.65	.785
	Bachelor’s	100	90.9	51	92.7	49	89.1		
	Master’s	7	6.4	3	5.6	4	7.3		
Position^†^	General nurse	108	98.2	54	98.2	54	98.2	0.00	1.000
	Charge nurse	2	1.8	1	1.8	1	1.8		
Total career (yr)^†^	＜ 2	20	18.2	10	18.2	10	18.2	3.29	.358
	2–4.9	33	30.0	19	34.6	14	25.5		
	5–9.9	51	46.4	25	45.5	26	47.3		
	≥ 10	6	5.5	1	1.8	5	9.1		
ICU career (yr)^†^	＜ 2	31	28.2	13	23.6	18	32.7	4.59	.204
	2–4.9	38	34.6	24	43.6	14	25.5		
	5–9.9	37	33.6	17	30.2	20	36.4		
	≥ 10	4	3.6	1	1.8	3	5.5		
Received enteral nutrition education	Yes	78	70.9	43	78.2	35	63.6	2.82	.093
	No	32	29.1	12	21.8	20	36.4		
Source of enteral nutrition education^‡^	Nursing school	51	49.0	29	53.7	22	44.0		
	Hospital education	27	26.0	11	20.4	16	32.0		
	Academic conference	1	1.0	1	1.9	0	0.0		
	Journal articles	3	2.9	1	1.9	2	4.0		
	Colleagues	20	19.2	11	20.4	9	18.0		
	Internet	2	1.9	1	1.9	1	2.0		

ICU = intensive care unit. ^†^Fisher’s exact test; ^‡^Multiple response.

**Table 2.** Perceptions, Knowledge, and Performance of Enteral Nutrition and Homogeneity Test (N = 110)

Variables	Total (n = 110)	Control group (n = 55)	Experimental group (n = 55)	Z	*p*
	M ± SD	M ± SD	M ± SD		
Perceptions	50.88 ± 8.13	50.49 ± 9.25	51.27 ± 6.89	-0.83	.409
Responsibility	18.05 ± 3.26	17.69 ± 3.24	18.42 ± 3.26	-1.17	.244
Knowledge	14.42 ± 3.96	14.80 ± 4.42	14.04 ± 3.44	1.01	.314
Documentation	18.41 ± 2.97	18.00 ± 3.19	18.82 ± 2.69	-1.45	.149
Knowledge	8.72 ± 1.85	8.69 ± 1.85	8.75 ± 1.88	-0.33	.745
Performance	60.79 ± 6.03	59.36 ± 6.46	62.22 ± 5.24	-2.62	.009

M = mean; SD = standard deviation.

**Table 3.** Comparison of the Mean Difference in Pre- and Post-Test Scores for the Perceptions, Knowledge, and Performance of Enteral Nutrition between the Control and Experimental Groups (N = 110)

Variables		Pre-test	Post-test	Difference (Post-test - Pre-test)	Z	*p*
		M ± SD	M ± SD	M ± SD		
Perceptions	Cont. (n = 55)	50.49 ± 9.25	52.49 ± 8.27	2.00 ± 5.57	-4.04	<.001
	Exp. (n = 55)	51.27 ± 6.89	59.16 ± 7.57	7.89 ± 7.95		
Responsibility	Cont. (n = 55)	17.69 ± 3.24	18.24 ± 2.89	0.55 ± 2.32	-3.04	.002
	Exp. (n = 55)	18.42 ± 3.26	20.60 ± 2.90	2.18 ± 3.21		
Knowledge	Cont. (n = 55)	14.80 ± 4.42	15.49 ± 4.20	0.69 ± 2.90	-4.57	<.001
	Exp. (n = 55)	14.04 ± 3.44	17.78 ± 3.33	3.75 ± 3.58		
Documentation	Cont. (n = 55)	18.00 ± 3.19	18.76 ± 2.82	0.76 ± 2.21	-2.25	.024
	Exp. (n = 55)	18.82 ± 2.69	20.78 ± 2.43	1.96 ± 2.99		
Knowledge	Cont. (n = 55)	8.69 ± 1.85	8.71 ± 2.03	0.02 ± 1.91	-7.48	<.001
	Exp. (n = 55)	8.75 ± 1.88	12.93 ± 2.25	4.18 ± 2.33		
Performance	Cont. (n = 55)	59.36 ± 6.46	59.42 ± 5.86	0.06 ± 3.96	-2.20	.028
	Exp. (n = 55)	62.22 ± 5.24	64.22 ± 4.47	2.00 ± 5.14		

M = mean; SD = standard deviation; Cont.= control group; Exp.= experimental group.

## Data Availability

Please contact the corresponding author for data availability.

## References

[1] McClave SA, Taylor BE, Martindale RG, Warren MM, Johnson DR, Braunschweig C (2016). Guidelines for the provision and assessment of nutrition support therapy in the adult critically ill patient: Society of Critical Care Medicine (SCCM) and American Society for Parenteral and Enteral Nutrition (A.S.P.E.N.). Journal of Parenteral and Enteral Nutrition.

[2] Lew CCH, Yandell R, Fraser RJ, Chua AP, Chong MFF, Miller M (2017). Association between malnutrition and clinical outcomes in the intensive care unit: a systematic review. Journal of Parenteral and Enteral Nutrition.

[3] Ahn S, Na SH, Chang CH, Lim H, Lee DC, Shin CS (2012). Effects of APACHE II score and initial nutritional status on prognosis of the critically ill patients. Acute and Critical Care.

[4] Elamin EM, Camporesi E (2009). Evidence-based nutritional support in the intensive care unit. International Anesthesiology Clinics.

[5] Cadena AJ, Habib S, Rincon F, Dobak S (2020). The benefits of parenteral nutrition (PN) versus enteral nutrition (EN) among adult critically ill patients: what is the evidence? a literature review. Journal of Intensive Care Medicine.

[6] Druml C, Ballmer PE, Druml W, Oehmichen F, Shenkin A, Singer P (2016). ESPEN guideline on ethical aspects of artificial nutrition and hydration. Clinical Nutrition.

[7] Marshall AP, Cahill NE, Gramlich L, MacDonald G, Alberda C, Heyland DK (2012). Optimizing nutrition in intensive care units: empowering critical care nurses to be effective agents of change. American Journal of Critical Care.

[8] Kim H, Stotts NA, Froelicher ES, Engler MM, Porter C (2012). Why patients in critical care do not receive adequate enteral nutrition? a review of the literature. Journal of Critical Care.

[9] Heyland DK, Cahill NE, Dhaliwal R, Sun X, Day AG, McClave SA (2010). Impact of enteral feeding protocols on enteral nutrition delivery: results of a multicenter observational study. Journal of Parenteral and Enteral Nutrition.

[10] Yun SH, Kim SJ, Oh EG (2012). Healthcare professional's knowledge, perception and performance on early enteral nutrition for critically ill patients. Korean Journal of Critical Care Medicine.

[11] Bjerrum M, Tewes M, Pedersen P (2012). Nurses’ self-reported knowledge about and attitude to nutrition–before and after a training programme. Scandinavian Journal of Caring Sciences.

[12] Kim H, Chang SJ (2019). Implementing an educational program to improve critical care nurses' enteral nutritional support. Australian Critical Care.

[13] Bedier NA, EL-Ata ABA, Shehab MS (2016). Effect of educational program on nurses' practice related to care of patients undergoing nasogastric tube feeding. International Journal of Caring Sciences.

[14] Ahmed AT, Hassan HB (2021). Interventional nursing program for nurses practices about enteral feeding guidelines in critical units. Indian Journal of Forensic Medicine & Toxicology.

[15] Byun GR, Park JE, Hong HS (2015). The effects of video programs of cardiopulmonary cerebral resuscitation education. Journal of Korean Biological Nursing Science.

[16] Kook MJ (2003). A study on the effect and application of motion picture materials in geography subject. Journal of Geographic and Environmental Education.

[17] Lee YS, Jeong HS (2019). The effect of fundamental nursing practicum method by using QRcode: focusing on intramuscular injection practice. Asia-pacific Journal of Multimedia Services Convergent with Art, Humanities, and Sociology.

[18] Cho KL, Kim SY (2018). Inquiry of learner’s experiences provided by smart phone learning environment based on Dewey’s theory of education. Journal of Educational Technology.

[19] Moon DH, Kim KH (2022). Effect of self-care education using a QR-Code on self-efficacy, self-care performance, and education satisfaction among discharged pneumothorax patients. Korean Journal of Adult Nursing.

[20] Jeon YJ (2015). A study on technology embedded English classes using QR codes. International Journal of Contents.

[21] Cho J, Seo GW, Lee JS, Cho HK, Kang EM, Kim J (2021). The usefulness of the QR code in orthotic applications after orthopedic surgery. Healthcare.

[22] Hu J, Ren J, Zheng J, Li Z, Xiao X (2020). A quasi-experimental study examining QR code-based video education program on anxiety, adherence, and satisfaction in coronary angiography patients. Contemporary Nurse.

[23] Persenius MW, Hall-Lord ML, Bååth C, Larsson BW (2008). Assessment and documentation of patients’ nutritional status: perceptions of registered nurses and their chief nurses. Journal of Clinical Nursing.

[24] Kim H, Soun E (2016). Critical care nurses’ perception, knowledge, and practices of enteral nutrition. Journal of Korean Academy of Fundamentals of Nursing.

[25] McClave SA, Martindale RG, Vanek VW, McCarthy M, Roberts P, Taylor B (2009). Guidelines for the provision and assessment of nutrition support therapy in the adult critically ill patient: Society of Critical Care Medicine (SCCM) and American Society of Parenteral and Enteral Nutrition (A.S.P.E.N.). Journal of Parenteral and Enteral Nutrition.

[26] Kreymann KG, Berger MM, Deutz NE, Hiesmayr M, Jolliet P, Kazandjiev G (2006). ESPEN guidelines on enteral nutrition: intensive care. Clinical Nutrition.

[27] Kim KH (2021). Development of a video-based critical care nutrition support education program for intensive care unit nurses [master’s thesis].

[28] Ebbinghaus H (2013). Memory: a contribution to experimental psychology. Annals of Neurosciences.

[29] Kim SS, Choi YS (2020). Effects of a repeated hemodialysis diet education program for older adults. Korean Journal of Adult Nursing.

[30] Ahmed FAHM, Ahmed OAE, Abd E, Albitar E, Ghoneim SES (2018). Effect of educational nursing guidelines regarding enteral feeding on nurses' knowledge and practices at critical care units. IOSR Journal of Nursing and Health Science.

[31] Bloomer MJ, Clarke AB, Morphet J (2018). Nurses' prioritization of enteral nutrition in intensive care units: a national survey. Nursing in Critical Care.

[32] Ameri ZA, Vafaee A, Sadeghi T, Mirlashari Z, Ghoddoosi-Nejad D, Kalhor F (2016). Effect of a comprehensive total parenteral nutrition training program on knowledge and practice of nurses in NICU. Global Journal of Health Science.

[33] Babapour SK, Esmaeili R, Esteki T, Naderiravesh N, Pourhoseingholi MA, Marzangu SMH (2016). Nurses’ practice about performance of nasogastric tube feeding in intensive care unit. International Journal of Advanced Biotechnology and Research.

[34] Björn A, Pudas-Tähkä SM, Salanterä S, Axelin A (2017). Video education for critical care nurses to assess pain with a behavioural pain assessment tool: a descriptive comparative study. Intensive and Critical Care Nursing.

